# A perioperative multi-modal fusion and deep learning-based prognostic system for upper tract urothelial carcinoma: a multi-institutional study

**DOI:** 10.1186/s13244-026-02337-x

**Published:** 2026-06-25

**Authors:** Xiang Peng, Yang Li, Wei Shi, Bangxin Xiao, Xiao Xiao, Xiaofeng Yue, Qiao Xv, Qing Jiang, Weiyang He, Yingjie Xv, Mingzhao Xiao

**Affiliations:** 1 Department of Urology, The First Affiliated Hospital of Chongqing Medical University, Chongqing, China; 2 Department of Urology, Urologic Surgery Center, Xinqiao Hospital, Third Military Medical University (Army Medical University), Chongqing, China; 3 Center for Reproductive Medicine, Women and Children’s Hospital of Chongqing Medical University, Chongqing Health Center for Women and Children, Chongqing, China; 4 Department of Urology, Chongqing University Fuling Hospital, Chongqing, China; 5 Department of Urology, The Third Affiliated Hospital of Chongqing Medical University, Chongqing, China; 6 Department of Urology, Yongchuan Hospital of Chongqing Medical University, Chongqing, China; 7 Department of Urology, The Second Affiliated Hospital of Chongqing Medical University, Chongqing, China

**Keywords:** Urothelial carcinoma, Computed tomography, Deep learning, Radiomics, Prognosis

## Abstract

**Objectives:**

Precise perioperative risk stratification for upper tract urothelial carcinoma (UTUC) is essential. We developed a multimodal prognostic model integrating perioperative clinical data, radiomics, and deep learning (DL) features from baseline CT urography to improve survival prediction and guide adjuvant management.

**Materials and methods:**

We retrospectively enrolled 623 patients from six institutions, divided into training, internal validation, and independent external validation sets. Four single-modal models (clinical, radiomics, 2D DL, and 2.5D DL) were developed, and an integrated combined model was constructed by fusing their prognostic scores. Performance was evaluated using the C-index, area under the curve (AUC), calibration curves, and decision curve analysis (DCA).

**Results:**

The combined model consistently outperformed all single-modal models across all cohorts. C-indices reached 0.758 (95% CI: 0.712–0.804), 0.725 (95% CI: 0.651–0.798), and 0.704 (95% CI: 0.631–0.777) in the training, internal validation, and external validation sets, respectively, numerically surpassing the best single-modal models. Notably, our 2.5D DL model (C-index: 0.705) demonstrated a consistent incremental improvement over the 2D DL model (C-index: 0.681) in capturing prognostic information. In external validation, the combined model achieved a 3-year AUC of 0.766. DCA indicated the comprehensive model exhibited excellent calibration and provided the highest net benefits.

**Conclusion:**

This multimodal system, featuring a robust 2.5D DL strategy, improves overall survival prediction in UTUC. It offers a valuable tool for accurate perioperative risk stratification immediately after radical nephroureterectomy, demonstrating particularly reliable value for 3-year intermediate-term clinical decision-making.

**Critical relevance statement:**

This multimodal system advances clinical radiology by fusing perioperative clinical data, radiomics, and DL features from CTU images, enhancing risk stratification accuracy to guide postoperative adjuvant management for UTUC.

**Key Points:**

- Current prognostic models for UTUC lack accuracy, creating an unmet clinical need for precise perioperative risk stratification to guide adjuvant management.
- A multimodal prognostic model fusing clinical, radiomic, and DL features from baseline CT urography consistently outperformed single-modality models in predicting overall survival.

**Graphical Abstract:**

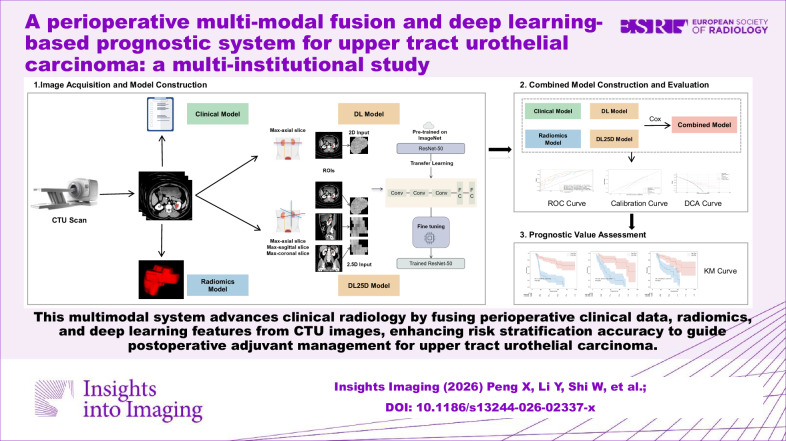

## Introduction

Upper urinary tract urothelial carcinoma (UTUC) is a relatively rare but aggressive malignancy originating in the renal pelvis and ureteral urothelium [1, 2]. Although it accounts for only 5%–10% of all urothelial carcinomas, UTUC is characterized by a high recurrence rate and rapid progression [1, 3–5]. While radical nephroureterectomy (RNU) remains the standard treatment for localized UTUC, accurate risk stratification is crucial for guiding perioperative management, particularly regarding the administration of adjuvant chemotherapy and the intensity of follow-up surveillance [1, 6]. However, current prognostic systems, which primarily rely on conventional clinicopathological features such as tumor stage and grade, are limited in their predictive ability and fail to fully capture the complexity and heterogeneity of the disease [7–9]. Furthermore, current preoperative staging based solely on CT (cT) is often inaccurate compared to pathological staging (pT) [1, 6], which may introduce substantial noise and limit the reliability of clinical decision-making. Consequently, there is an urgent clinical need for a more comprehensive and accurate prognostic model.

Recently, artificial intelligence (AI), including radiomics and deep learning (DL), has opened up promising new avenues for medical prognosis by decoding tumor heterogeneity [10–12]. Radiomics extracts high-throughput quantitative features from standard medical images, while DL can automatically learn high-dimensional feature patterns directly from original data [13–16]. These technologies allow for the identification of profound image features closely associated with clinical prognosis that are often difficult to discern through visual inspection [17–19]. The deep integration of radiomics and DL offers a potent technical tool for constructing a more precise and quantifiable prognostic model for UTUC, which is hypothesized to overcome the current impasse in clinical risk assessment.

Despite these advancements, existing UTUC prognostic research often relies on single-modal approaches. It must be explicitly clarified that applying multiple algorithms—such as both radiomics and DL—exclusively to CT images still fundamentally constitutes a single-modality approach. Such single-source imaging strategies inherently lack the capacity to capture the complete clinical landscape. We posit that a true multimodal framework, which integrates these high-dimensional imaging phenotypes with complementary clinical and pathological data, can provide biological information that a single source may not capture [20, 21]. The synergistic integration of multimodal data is essential for a comprehensive understanding of tumor behavior. Notable progress has been made in specific oncological fields by integrating multimodal data [22, 23]. For example, in their research on pediatric low-grade gliomas (pLGGs), Mahootiha et al [24] constructed a comprehensive multimodal DL model that significantly improved the accuracy of predicting postoperative recurrence by combining traditional clinical indicators with DL features automatically extracted from preoperative MRI scans. Therefore, the primary goal of this study is to construct a robust multimodal fusion model.

However, the performance of such a multimodal model depends heavily on the quality of feature extraction from the imaging modality. In this study, we constructed a combined prognostic model by integrating perioperative clinical data, radiomic features, and DL signatures, which markedly enhanced the accuracy of overall survival (OS) prediction in UTUC. To enhance the imaging component within this fusion framework, we introduce an innovative 2.5D multi-view DL strategy. This strategy effectively captures multiplanar information and reveals tumor heterogeneity more comprehensively than traditional 2D slice analysis. Through a large-scale, multicenter cohort study, we rigorously validated the model’s robustness and generalizability, fully demonstrating its potential for perioperative clinical application.

## Methods

### Patients and study design

**Fig. 1:**
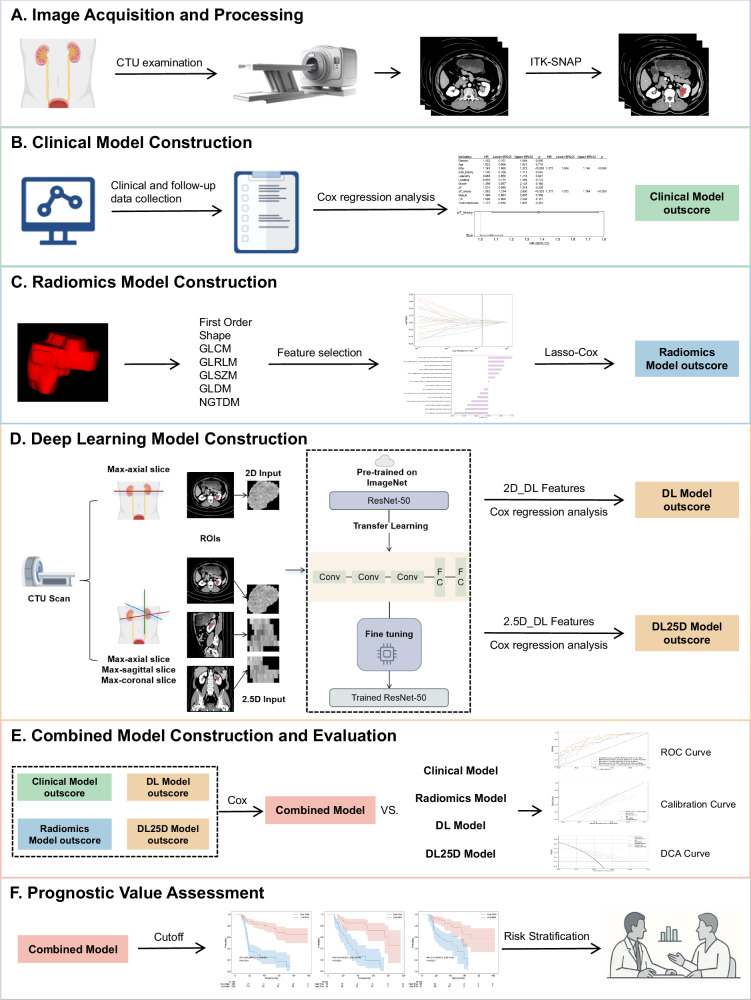
Overall workflow schematic. The comprehensive workflow for predicting the OS of patients with UTUC using CT images is shown. **A** Image acquisition and processing involved collecting baseline CTU scans, segmenting ROIs using ITK-SNAP software. **B** Clinical features were collected and a clinical model was constructed using Cox regression analysis. **C** The radiomics model was built by extracting first-order, shape, and texture features, followed by feature selection using Lasso-Cox regression analysis. **D** The DL model and DL25D model were constructed using a pre-trained ResNet-50 network for feature extraction from 2D and 2.5D CT inputs, respectively, followed by Cox regression analysis. **E** The combined model was established by integrating the clinical, radiomics, and DL model scores using Cox regression. The performance of all models was evaluated using ROC curves, calibration curves, and DCA. **F** The prognostic value of the final Combined Model was assessed by stratifying patients into high- and low-risk groups based on a cutoff value. OS, overall survival; UTUC, upper tract urothelial carcinoma; CTU, computed tomography urography; ROIs, regions of interest; DL, deep learning; ROC, receiver operating characteristic; DCA, decision curve analysis.

This study was ethically approved (CY2024-308-02) with informed consent waived. In this study, perioperative risk stratification is explicitly defined as the risk assessment performed immediately following RNU. At this clinical time point, the model integrates baseline preoperative imaging features with the newly available postoperative pathological staging to provide a comprehensive evaluation for subsequent adjuvant management. The overall workflow of this study is illustrated in Fig. 1. This multicenter retrospective study was approved by the Ethics Committee of our institutions, and the requirement for informed consent was waived; it was conducted in accordance with the Declaration of Helsinki. This study follows the CLEAR checklist guidelines [25]. A total of 623 patients with pathologically confirmed upper tract urothelial carcinoma (UTUC) who underwent RNU between December 2012 and December 2024 were included. Inclusion criteria were as follows: (1) histologically confirmed UTUC and (2) availability of preoperative computed tomography urography (CTU) data. Patients were excluded if they had poor image quality, received prior oncologic treatment before CTU, had a coexisting primary malignancy, or lacked complete clinicopathological or follow-up data. The detailed patient selection process is illustrated in Fig. S1. Patients from Centers 1 and 2 (*n* = 473) were randomly divided into a training set (*n* = 331) and an internal validation set (*n* = 142) at a 7:3 ratio. For independent validation, an external validation set was composed of 150 patients from four other centers (Centers 3, 4, 5, and 6). The primary endpoint of this study was OS, defined as the time from the initial diagnosis to death from any cause. Patients with limited follow-up duration (e.g., those enrolled in 2024) were included in the risk set, and their survival data were validly treated as censored at their last follow-up date.

### Image acquisition and preprocessing

Preoperative CTU images were retrieved in digital imaging and communications in medicine (DICOM) format from the Picture Archiving and Communication System for each institution. Acquisition protocols from the six centers are summarized in Table S1. To ensure consistency across institutions, the corticomedullary phase images were consistently selected for analysis, as this phase provided optimal tumor-to-background contrast. To minimize inter-scanner variability, all images underwent a standardized preprocessing pipeline: (1) Voxel spacing normalization: All CTU images were resampled to an isotropic voxel spacing of 1 × 1 × 1 mm³ using cubic interpolation. (2) Intensity normalization and contrast adjustment: The window level and width were standardized to 50 HU and 200 HU (range: −50 to 150 HU). (3) Region of interest (ROIs) segmentation: The Regions of Interest (ROIs) were manually delineated on the axial CT slices. To ensure segmentation precision, all delineations were uniformly performed on the corticomedullary phase images. This segmentation process was conducted by two experienced radiologists using ITK-SNAP software. Discrepancies were resolved by a senior radiologist (20 years of experience). To quantitatively assess inter-observer reproducibility, 30 cases were randomly selected and re-segmented independently by the second radiologist to calculate the intraclass correlation coefficient (ICC, two-way mixed-effects model, single rater, absolute agreement).

### Clinical and radiomics model construction

Clinical variables, including age, sex, tumor size, pT stage, grade, lymphovascular invasion (LVI), and hydronephrosis, were extracted from medical records. Continuous variables were tested for normality (Shapiro–Wilk test) and compared using the *t*-test or Mann–Whitney *U*-test; categorical variables were compared using the Chi-square (χ^2^) test. In our analysis, the pathologic T stage (pT) was further simplified into a binary variable, pT_binary, where patients with stages T0/T*x*/T1 were classified as non-muscle invasive (< T2), and those with stages T2, T3, and T4 were classified as muscle-invasive (≥ T2). This binary classification was chosen because the distinction between organ-confined and muscle-invasive disease is a critical clinical determinant for adjuvant chemotherapy and lymph node dissection indications [1].

Radiomic features were extracted from segmented ROIs using PyRadiomics (v3.0.1) following the Imaging Biomarker Standardization Initiative (IBSI) guidelines. Prior to feature reduction, *Z*-score normalization was applied to standardize the distribution of the extracted radiomics features. After voxel resampling and intensity discretization (64 gray levels), seven feature classes were computed: first-order, shape, GLCM, GLRLM, GLSZM, GLDZM, and NGTDM. Initially, 1288 radiomics features were extracted from the defined ROIs. The four-step feature reduction process yielded the following feature counts: (1) reproducibility filtering retained 864 features with ICC ≥ 0.85; (2) correlation filtering removed highly redundant variables, reducing the pool to 187 features; (3) univariate Cox analysis identified 28 features significantly associated with OS (*p* < 0.05); and (4) Finally, LASSO-Cox regression with 10-fold cross-validation selected an optimal subset of 15 features to construct the final radiomic signature (Fig. S2).

### DL model construction

The DL models were developed using a transfer learning approach.

#### Data preparation

To prepare the data, we adopted two distinct input strategies. For the DL (2D) model, we used the max-axial slice of the tumor as the representative input. For the DL25D (2.5D) model, a multi-view ROI extraction strategy was adopted, where the max-axial slice, max-sagittal slice, and max-coronal slice were extracted from each 3D tumor volume and stacked to form a multi-channel input (Fig. 1D). To ensure spatial alignment and preserve high-resolution intratumoral heterogeneity across different network architectures, the extracted ROIs were cropped and resized to 448×448 pixels using bicubic interpolation. Image intensities were subsequently Z-score normalized to standard deviation units. This 2.5D approach was designed to integrate global cross-sectional multiplanar features and simultaneously mitigate the memory limitations and severe overfitting risks associated with full 3D processing or adjacent-slice sequence modeling on clinical datasets.

#### Model architecture and transfer learning

We tested four well-known architectures—ResNet50, Vgg19, DenseNet121, and Inception_v3—using weights that had already been trained on ImageNet. The initial convolutional layer was reconfigured to receive multi-channel inputs, and the late-stage features were fed into a fully connected layer with L2 regularization, followed by a single-node output for log-risk prediction. The network was trained using the Cox partial likelihood loss to model non-linear relationships between imaging-derived features and survival outcomes [26]. Detailed hyperparameter configurations, including the learning rate scheduler, optimization strategies, and regularization techniques, are provided in Supplementary materials.

### Model interpretability

To offer commentary on the model’s decision-making process, we used gradient-weighted class activation mapping (Grad-CAM) to generate heatmaps [27]. These visualizations highlight the specific tumor regions that were most influential for the model’s prognostic predictions.

### Statistical analysis and model evaluation

All data analyses were executed using Python (v3.7.12) on the OnekeyAI platform. Our DL frameworks were developed on PyTorch (v1.11.0), optimized for enhanced performance through CUDA (v11.3.1) and cuDNN (v8.2.1).

The prognostic scores derived from each optimized single-modality model were entered into a multivariate Cox regression with stepwise selection to construct the final combined prognostic model. For survival analysis, Kaplan–Meier (KM) curves were generated, and samples were stratified based on the predicted hazard ratios (HRs), with the significance of group separation assessed via the log-rank test. The discrimination performance of the models was evaluated using the Concordance index (C-index) and time-dependent area under the curve (AUC). To robustly assess internal and external validity, 95% confidence intervals (CIs) for the metrics were calculated using bootstrap resampling with 1000 iterations. Calibration was evaluated using calibration curves and quantified by the Integrated Brier Score, reflecting the agreement between predicted and observed survival probabilities. Furthermore, decision curve analysis (DCA) was utilized to evaluate the clinical net benefit of the models across various threshold probabilities.

## Results

### Patient characteristics and cohort distribution

As shown in Table 1, a total of 623 patients with UTUC were enrolled from six centers and divided into a training cohort (*n* = 331), an internal validation cohort (*n* = 142), and an external validation cohort (*n* = 150). The mean age of all enrolled patients was 68.21 years (± 10.06). The majority of patients were male (58.9%) and had a tumor size greater than or equal to 2 cm (74.0%). The baseline clinical and pathological characteristics were statistically similar across the three cohorts, with no significant differences found in key variables such as age, gender, tumor size, laterality, location, grade, margin, LVI, and hydronephrosis (*p* > 0.05). However, significant differences were observed in pT stage (*p* = 0.038) and pT_binary (*p* = 0.006). The median follow-up duration for the entire cohort was 24.03 months (IQR: 13.68–32.28).

**Table 1:** Baseline characteristics of UTUC patients

Characteristic	All patients	Training set	Internal validation set	External validation set	*p*
	(*n* = 623)	(*n* = 331)	(*n* = 142)	(*n* = 150)	
Age	68.212 ± 10.058	67.644 ± 10.204	68.634 ± 9.794	69.067 ± 9.966	0.303
Gender					0.782
Female	256 (41.1%)	138 (41.7%)	60 (42.3%)	58 (38.7%)	
Male	367 (58.9%)	193 (58.3%)	82 (57.7%)	92 (61.3%)	
Size (cm)	3.142 ± 2.080	3.092 ± 1.922	2.995 ± 1.799	3.393 ± 2.595	0.214
Size_binary					0.443
< 2 cm	162 (26.0%)	93 (28.1%)	33 (23.2%)	36 (24.0%)	
≥ 2 cm	461 (74.0%)	238 (71.9%)	109 (76.8%)	114 (76.0%)	
Laterality					0.350
Left	342 (54.9%)	189 (57.1%)	78 (54.9%)	75 (50.0%)	
Right	281 (45.1%)	142 (42.9%)	64 (45.1%)	75 (50.0%)	
Location					0.122
Pelvis	284 (45.6%)	144 (43.5%)	75 (52.8%)	65 (43.3%)	
Ureter	303 (48.6%)	168 (50.8%)	63 (44.4%)	72 (48.0%)	
Both	36 (5.8%)	19 (5.7%)	4 (2.8%)	13 (8.7%)	
Grade					0.687
High	496 (79.6%)	262 (79.2%)	111 (78.2%)	123 (82.0%)	
Low	127 (20.4%)	69 (20.8%)	31 (21.8%)	27 (18.0%)	
pT stage					0.038
Tx/T0	83 (13.2%)	52 (15.7%)	20 (14.1%)	11 (7.3%)	
T1	182 (29.2%)	101 (30.5%)	45 (31.7%)	36 (24.0%)	
T2	176 (28.3%)	92 (27.8%)	41 (28.9%)	43 (28.7%)	
T3	163 (26.2%)	78 (23.6%)	33 (23.2%)	52 (34.7%)	
T4	19 (3.1%)	8 (2.4%)	3 (2.1%)	8 (5.3%)	
pT_binary					0.006
< T2	265 (42.5%)	153 (46.2%)	65 (45.8%)	47 (31.3%)	
≥ T2	358 (57.5%)	178 (53.8%)	77 (54.2%)	103 (68.7%)	
Margin					0.337
Negative	598 (96.0%)	315 (95.2%)	136 (95.8%)	147 (98.0%)	
Positive	25 (4.0%)	16 (4.8%)	6 (4.2%)	3 (2.0%)	
LVI					0.504
No	557 (89.4%)	296 (89.4%)	130 (91.5%)	131 (87.3%)	
Yes	66 (10.6%)	35 (10.6%)	12 (8.5%)	19 (12.7%)	
Hydronephrosis					0.577
No	277 (44.5%)	145 (43.8%)	60 (42.3%)	72 (48.0%)	
Yes	346 (55.5%)	186 (56.2%)	82 (57.7%)	78 (52.0%)	
Median follow-up, months	24.03 (13.68 - 32.28)	23.18 (13.26–29.98)	17.63 (13.54–30.81)	29.90 (15.59–36.08)	0.086

### Performance of clinical and radiomics models

**Fig. 2:**
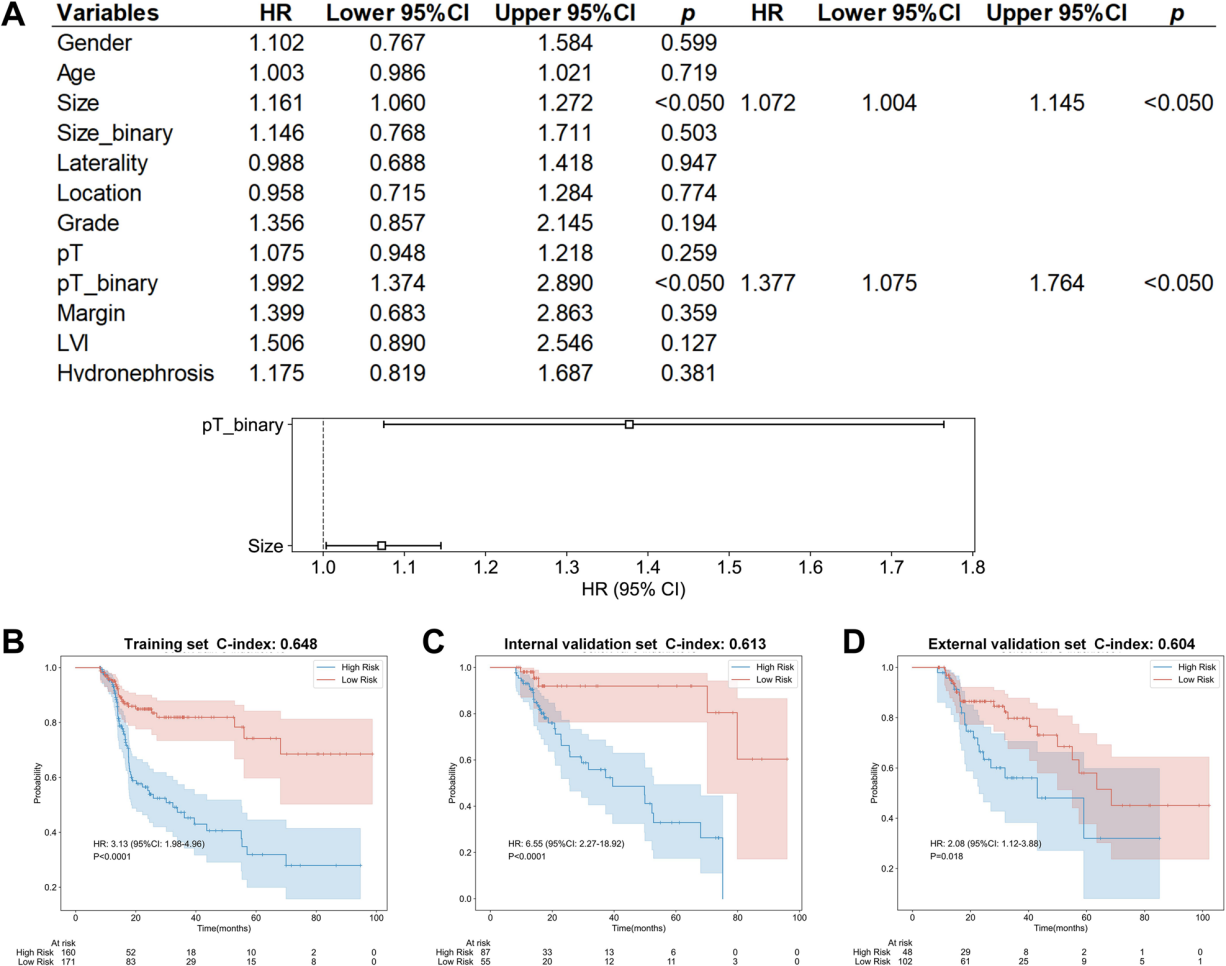
Construction and prognostic value of the clinical model. **A** Univariate and multivariate Cox regression analysis was performed to identify significant clinical features for the clinical model. The forest plot shows the HR and 95% CI for each variable. **B**–**D** KM survival curves for the Clinical Model are shown in the training set (**B**), internal validation set (**C**), and external validation set (**D**). HR, hazard ratios; CI, confidence intervals; KM, Kaplan–Meier

We first evaluated the predictive power of two single-modal models. The clinical model, constructed using multivariate Cox regression (Fig. 1B), identified pT_binary and tumor size as significant prognostic factors (Fig. 2A). This model achieved a C-index of 0.648 (95% CI: 0.596–0.699) in the training cohort and was validated with C-indexes of 0.613 (95% CI: 0.533–0.694) and 0.604 (95% CI: 0.525–0.682) in the internal and external validation sets, respectively (Fig. 2B–D and Table 2).

**Table 2:** The performance of models in predicting OS in UTUC patients

Cohorts	Models	*C*-index	1-year AUC	3-year AUC	5-year AUC
Training set	Clinical model	0.648 (0.596–0.699)	0.518 (0.392–0.643)	0.699 (0.612–0.786)	0.743 (0.641–0.844)
	Radiomics model	0.664 (0.614–0.715)	0.542 (0.406–0.679)	0.750 (0.667–0.832)	0.798 (0.693–0.904)
	DL model	0.681 (0.631–0.731)	0.617 (0.493–0.741)	0.755 (0.674–0.837)	0.792 (0.674–0.909)
	DL25D model	0.705 (0.656–0.754)	0.678 (0.562–0.793)	0.782 (0.706–0.858)	0.843 (0.750–0.937)
	Combined model	0.758 (0.712–0.804)	0.688 (0.594–0.783)	0.842 (0.776–0.907)	0.887 (0.803–0.971)
Internal validation set	Clinical model	0.613 (0.533–0.694)	0.528 (0.312–0.743)	0.645 (0.499–0.792)	0.689 (0.504–0.873)
	Radiomics model	0.656 (0.578–0.734)	0.726 (0.541–0.911)	0.732 (0.598–0.866)	0.690 (0.544–0.837)
	DL model	0.656 (0.578–0.734)	0.764 (0.581–0.946)	0.623 (0.475–0.771)	0.595 (0.418–0.773)
	DL25D model	0.668 (0.591–0.746)	0.809 (0.705–0.913)	0.738 (0.603–0.873)	0.756 (0.618–0.894)
	Combined model	0.725 (0.651–0.798)	0.830 (0.738–0.921)	0.755 (0.627–0.883)	0.782 (0.647–0.917)
External validation set	Clinical model	0.604 (0.525–0.682)	0.664 (0.400–0.927)	0.703 (0.578–0.829)	0.678 (0.506–0.850)
	Radiomics model	0.657 (0.581–0.733)	0.785 (0.591–0.979)	0.731 (0.613–0.849)	0.711 (0.497–0.925)
	DL model	0.651 (0.575–0.727)	0.863 (0.663–1.000)	0.630 (0.499–0.761)	0.597 (0.387–0.806)
	DL25D model	0.658 (0.582–0.734)	0.704 (0.385–1.000)	0.664 (0.535–0.793)	0.702 (0.512–0.891)
	Combined model	0.704 (0.631–0.777)	0.818 (0.620–1.000)	0.766 (0.653–0.880)	0.760 (0.575–0.945)

**Fig. 3:**
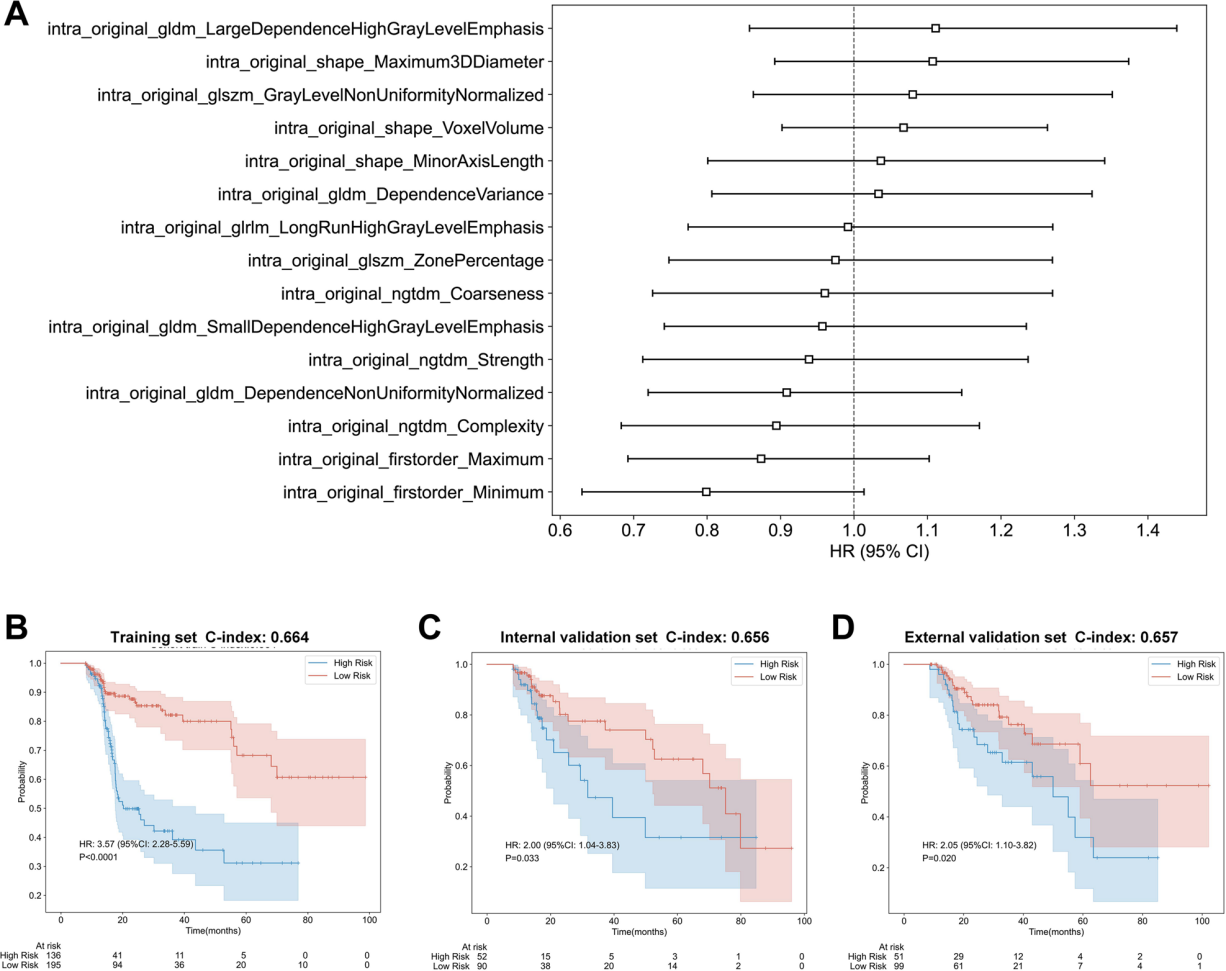
Construction and prognostic value of the radiomics model. **A** A forest plot shows the HR and 95% CI of the selected radiomics features, which were identified using Lasso-Cox regression analysis. **B**–**D** KM survival curves for the Radiomics Model are shown in the training set (**B**), internal validation set (**C**), and external validation set (**D**). HR, hazard ratios; CI, confidence intervals; KM, Kaplan–Meier

During the construction of the radiomics model, we adopted a standardized workflow that included the extraction of features from three sets of dimensions: first-order, shape, and texture features (Figure S2). Following feature selection using LASSO-Cox regression, the KM survival curves of the final model demonstrated the predictive performance, with C-indexes of 0.664 (95% CI: 0.614–0.715), 0.656 (95% CI: 0.578–0.734), and 0.657 (95% CI: 0.581–0.733) in the three study cohorts, respectively (Fig. 3 and Table 2).

### Optimization and performance of DL models

**Fig. 4:**
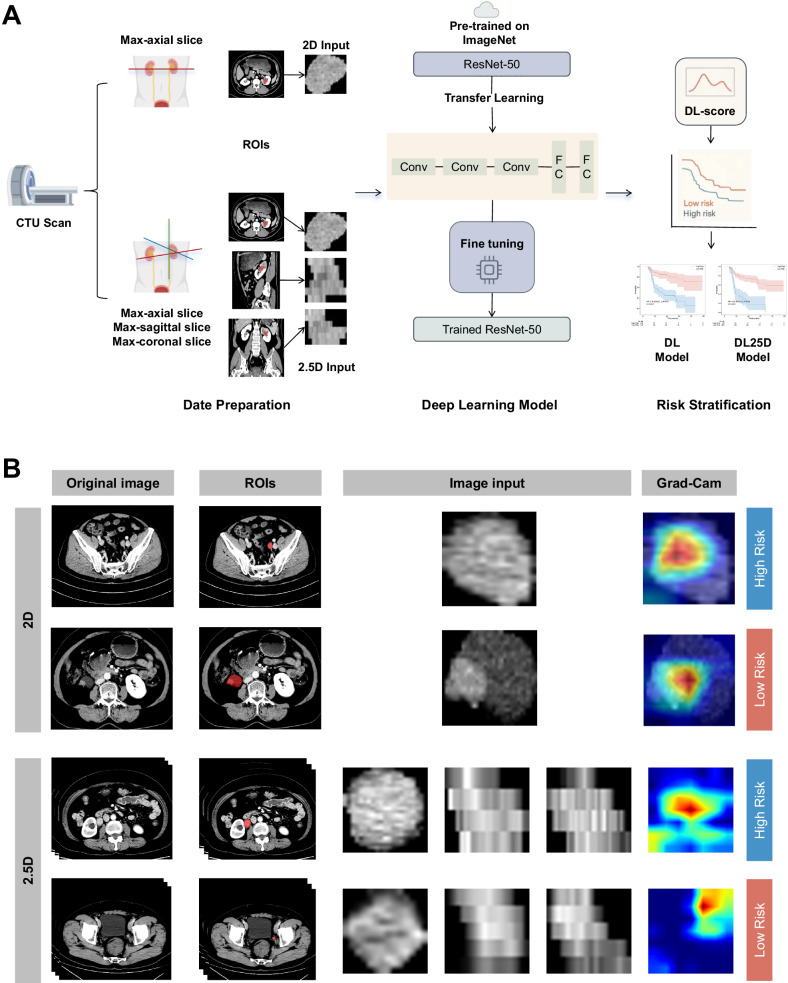
Workflow of DL models and Grad-CAM visualization. **A** The framework for building the DL Model and DL25D Model is illustrated. Both models were based on a pre-trained ResNet-50 network, with the DL Model using 2D image inputs and the DL25D Model using 2.5D image inputs to capture multi-plane information. The models were fine-tuned to extract prognostic features (DL-score) for risk stratification. **B** Representative images and Grad-CAM heatmaps are shown for both 2D and 2.5D models in high-risk and low-risk patients. DL, deep learning; Grad-CAM, gradient-weighted class activation mapping

To identify the most effective DL architecture, we evaluated the prognostic performance of four popular networks on both 2D and 2.5D inputs (Fig. 4A). As detailed in Table S2, the 2D ResNet-50 model achieved a C-index of 0.681 in the training cohort, whereas the 2.5D ResNet-50 model achieved a modestly higher C-index of 0.705. Given its enriched spatial feature representation, the ResNet-50 architecture was selected for both 2D and 2.5D feature extraction.

The prognostic performance of the models based on these extracted features was then evaluated. As shown in Table 2, the DL model (based on 2D features) demonstrated C-indexes of 0.681 (95% CI: 0.631–0.731), 0.656 (95% CI: 0.578–0.734), and 0.651 (95% CI: 0.575–0.727) in the training, internal, and external validation sets, respectively (Fig. S3A). The DL25D model (based on 2.5D features) demonstrated C-indexes of 0.705 (95% CI: 0.656–0.754), 0.668 (95% CI: 0.591–0.746), and 0.658 (95% CI: 0.582–0.734) in the three cohorts, respectively (Fig. S3B). The decision-making process of both DL models was further visualized using Grad-CAM, which highlighted the tumor regions most crucial for their predictions (Fig. 4B).

### Construction and performance of the combined model

**Fig. 5:**
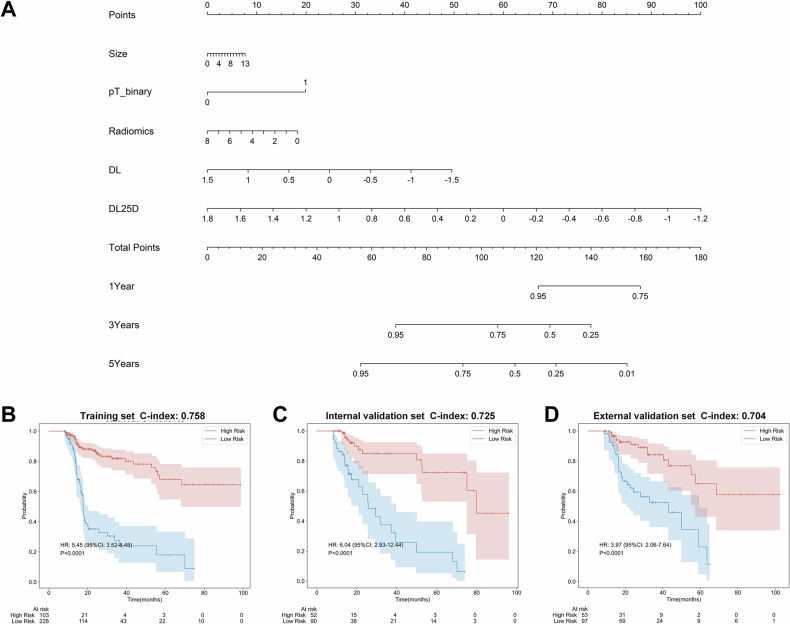
Development and prognostic validation of the combined model. **A** A nomogram for the combined model is presented, which integrates clinical (e.g., Size, pT_binary), radiomics, and DL features to predict 1-, 3-, and 5-year OS. **B**–**D** KM survival curves for the combined model are shown in the training set (**B**), internal validation set (**C**), and external validation set (**D**). DL, deep learning; KM, Kaplan–Meier

The prognostic scores from the clinical, radiomics, DL, and DL25D models were integrated to construct a comprehensive combined model, which served as a final prognostic system presented as a nomogram (Fig. 5A). This combined model consistently exhibited higher discrimination compared to all single-modal models across all cohorts. As presented in Table 2, the combined model achieved the highest C-index values of 0.758 (95% CI: 0.712–0.804), 0.725 (95% CI: 0.651–0.798), and 0.704 (95% CI: 0.631–0.777) in the training set, internal validation set, and external validation set, respectively. The robust prognostic capacity of the model was further corroborated by KM survival analysis, which disclosed significant disparities in OS between the high-risk group and the low-risk group across all three cohorts (Fig. 5B–D).

To further evaluate the prognostic robustness of the combined model across different tumor stages, a stratified analysis was performed based on the pathological T (pT) stage. Patients in each sub-cohort were stratified into high- and low-risk groups using the global median risk score derived from the training cohort. As shown in Figure S6, the combined model successfully discriminated survival outcomes in both the non-muscle invasive (< T2) and muscle-invasive (≥ T2) subgroups within the training and internal validation cohorts (all log-rank *p* < 0.05). In the independent external validation cohort, the model maintained significant risk stratification for the muscle-invasive (≥ T2) patients (log-rank *p* = 0.011). For the external early-stage (< T2) patients, although the survival difference was marginally significant (*p* = 0.066), likely due to the limited sample size and low event rate, a consistent trend of risk separation was observed. These results suggest that the combined model provides consistent prognostic information independent of the baseline pT stage.

### Comprehensive performance evaluation

**Fig. 6:**
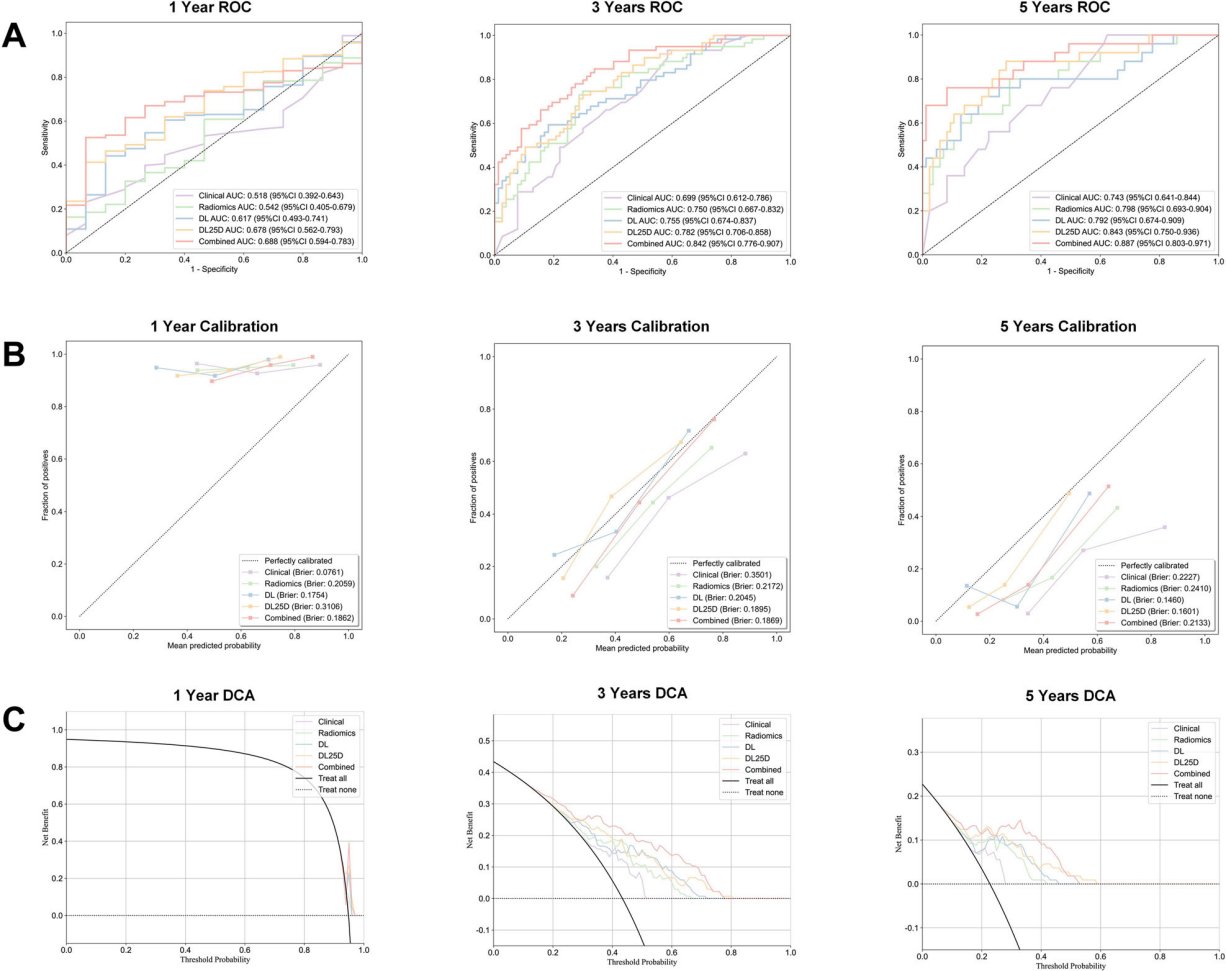
Model performance evaluation in the training set. This figure shows the performance of the Clinical, Radiomics, DL, DL25D, and Combined models within the training cohort. The performance of each model was evaluated at 1-, 3-, and 5-year follow-up intervals. **A** ROC curves are presented for all models. The AUC and 95% CI for each model are listed, demonstrating their discriminative ability. **B** Calibration curves are shown to assess the agreement between the predicted and actual survival probabilities. The dashed line represents a perfectly calibrated model. **C** DCA is presented to evaluate the clinical utility of each model. ROC, receiver operating characteristic; AUC, area under the ROC curve; CI, confidence interval; DCA, decision curve analysis

Lastly, ROC curve analysis, calibration analysis, and DCA were employed to comprehensively assess the performance of all models at 1, 3, and 5 years. As shown in Figs. 6, S4, and S5, the combined model exhibited the highest AUC values, suggesting superior discriminatory ability. For example, in the external validation set, the 3-year AUC value of the combined model attained 0.766 (95% CI: 0.653–0.880), numerically surpassing that of the clinical model (0.703), radiomics model (0.731), DL model (0.630), and DL25D model (0.664). The calibration curve of the combined model indicated a strong association between the predicted survival probability and the actual survival probability (e.g., Brier score = 0.187 at 3—year in the training set). The findings of the DCA revealed that the combined model consistently offered the highest net benefits compared to the single-modality models across a wide spectrum of probability thresholds, highlighting its clinical utility for adjuvant decision-making.

## Discussion

In this study, we developed and validated a combined prognostic model for patients with UTUC by integrating clinical, radiomics, and DL features. Our findings demonstrate that this combined model holds a substantial advantage over single-modal models, exhibiting superior discrimination ability, calibration performance, and clinical utility across the training, internal validation, and independent external validation sets. This prediction system provides a robust tool for personalized perioperative risk stratification immediately after RNU. By offering highly reliable 3-year intermediate-term survival predictions, it effectively guides adjuvant management strategies for UTUC patients.

Our findings provide compelling evidence that integrating multiple data modalities is an effective strategy for enhancing prognostic prediction. As shown in Table 2, the combined model achieved the highest C-index across all cohorts, indicating its robust performance. This conclusion is consistent with a growing consensus within the oncology community that multimodal models, which incorporate both clinical and imaging data, are more effective in predicting patient prognosis [28, 29]. This enhanced performance is underpinned by the complementary value between different data types. Clinical models provide macroscopic insights into patient status and tumor characteristics, such as pT stage and tumor size, which are established prognostic indicators [1, 30]. In contrast, radiomics features offer a quantitative lens to uncover tumor heterogeneity and provide profound insights into tumor biology and the microenvironment, which are difficult to discern through visual inspection alone [11, 13]. Previous studies have confirmed that specific radiomics features can serve as noninvasive biomarkers to predict gene mutations and treatment response [31, 32]. Additionally, DL features can automatically capture complex and subtle patterns in raw image data, including spatial associations—details that effectively complement standard radiomics profiling [33, 34]. By treating this integration as a cohesive analytical framework, our comprehensive combined model constructs a more complete representation of tumor characteristics.

While the overarching goal of this study is multimodal fusion, our core technical advancement lies in employing a 2.5D DL strategy to maximize the quality of features extracted from the imaging modality. Given the limited sample sizes typical in clinical research, full 3D DL models are prone to overfitting and require a substantial number of training samples for robust generalization. Our 2.5D model offers a practical middle ground, synthesizing maximum axial, sagittal, and coronal information to provide vital multiplanar context while reducing computational costs. As demonstrated in Table 2, the C-index of our 2.5D ResNet-50 model demonstrated a modest but consistent incremental improvement over the 2D model across all three cohorts. This synthesis allows the model to characterize tumor morphology, boundaries, and internal structures more precisely. Previous research has also shown the benefits of employing 2.5D or 3D convolutional neural networks for tasks like lung nodule classification and brain tumor segmentation, confirming their ability to enhance performance by leveraging volumetric data [35–37]. Moreover, model interpretability is crucial for clinical applications. The visualization results from Grad-CAM underscore that tumor margins and internal heterogeneity are key determinants of predictive risk, aligning well with established pathological prognostic factors [25, 38].

The most significant contribution of our study is the rigorous validation of our model across multi-institutional cohorts and varying disease severities. As shown in Table 1, significant differences in pT stage were observed across the cohorts—a common challenge in multi-institutional research [39, 40]. To explicitly address this imbalance and justify the incremental value of our imaging features beyond basic clinical staging, we conducted a rigorous subgroup analysis based on pT stage. Using a strict global median risk cutoff derived from the training cohort, the combined model successfully stratified patients into distinct prognostic groups within both the non-muscle invasive (< T2) and muscle-invasive (≥ T2) sub-cohorts (Fig. S6). Notably, in the unseen external validation cohort, the model maintained its robust stratification ability for muscle-invasive patients. For the external early-stage (< T2) subgroup, although the survival difference was marginally significant (*p* = 0.066) due to the limited sample size and the inherently low event rate, a strong clinical trend of risk separation was still evident. DCA depicted in Figs. 6, S4, and S5 further emphasize the clinical utility of our model. In the context of UTUC perioperative management, adopting the model’s recommendation (“treat”) implies implementing more intensive postoperative surveillance or considering adjuvant therapies for high-risk patients, whereas “treat none” implies standard routine follow-up. The DCA data indicate that our combined model offers a higher net benefit than default strategies across a broad spectrum of probability thresholds, aiding physicians in developing tailored follow-up plans.

Despite these promising results, our study has several limitations. First, while our study included a large multicenter cohort, all data were retrospectively collected. CT urography protocols (e.g., scanning phases, slice thicknesses) varied across institutions, and although images were resampled, inherent variations and potential interpolation artifacts may still influence feature extraction. Second, due to the retrospective nature of this real-world multicenter study, detailed postoperative treatment information (e.g., adjuvant chemotherapy) and clinical N stage were inconsistently documented across the majority of participating institutions and thus could not be incorporated into the current model. Consequently, we cannot completely exclude the possibility that the predicted survival outcomes might be partially confounded by variations in subsequent clinical management, alongside the inherent biological risk of the tumor. Third, our inclusion period extended to December 2024, meaning recently enrolled patients had limited follow-up, which directly impacted the stability of our long-term predictions. While our model demonstrated robust overall predictive ability, its calibration varied across time points. The slight deviation observed in the 1-year calibration curve is primarily attributable to the inherently low short-term postoperative mortality rate of UTUC; this extreme scarcity of positive events causes the model to lean towards predicting excessively high survival probabilities. Conversely, the instability in 5-year predictions is a direct consequence of the heavy right-censoring mentioned above. Because we did not impose a strict minimum follow-up duration in order to maximize the sample size for DL, this extreme tail-end sample attrition at the 5-year mark leads to wider CIs and less reliable long-term predictions. Fourth, regarding the 2.5D strategy, selecting only the maximum-area slices from three orthogonal planes captures global macroscopic morphology but may not fully encapsulate the continuous longitudinal spread of UTUC compared to slab-based (adjacent slices) or full 3D models. Finally, our model was designed to predict OS. Integrating other endpoints, such as cancer-specific survival (CSS) or recurrence-free survival (RFS), could provide a more comprehensive evaluation of patient prognosis in future prospective studies.

## Supplementary information

ELECTRONIC SUPPLEMENTARY MATERIAL

## Data Availability

The original data are not publicly available, but are available from the corresponding author upon reasonable request after IRB approval in accordance with the institute’s policy.

## Abbreviations

AUC: Area under the ROC curve
CI: Confidence interval
C-index: Concordance index
CTU: Computed tomography urography
DCA: Decision curve analysis
DL: Deep learning
HR: Hazard ratios
ICC: Intraclass correlation coefficient
KM: Kaplan–Meier
LVI: Lymphovascular invasion
OS: Overall survival
pT: Pathologic T stage
RNU: Radical nephroureterectomy
ROC: Receiver operating characteristic
ROI: Region of interest
UTUC: Upper urinary tract urothelial carcinoma
