# Supplementary material for: A perioperative multi-modal fusion and deep learning-based prognostic system for upper tract urothelial carcinoma: a multi-institutional study
Source: Insights Imaging. 2026 Jun 25;17:173. doi: 10.1186/s13244-026-02337-x (PMC13294415; doi:10.1186/s13244-026-02337-x)
Supplement: Supplementary file 1 — ELECTRONIC SUPPLEMENTARY MATERIAL [file 13244_2026_2337_MOESM1_ESM.pdf]

A Perioperative Multi-modal Fusion and Deep Learning-based Prognostic System for Upper Tract Urothelial Carcinoma: A Multi-institutional Study

ELECTRONIC SUPPLEMENTARY MATERIAL

Methods

Deep Learning Training Protocol:

Model Initialization and Transfer Learning

The deep learning models (ResNet50, Vgg19, DenseNet121, and Inception_v3) were developed using a transfer learning approach. To accelerate convergence and compensate for the limited medical imaging dataset size, the models were initialized with weights pre-trained on the ImageNet dataset. Input images were standardized using the ImageNet normalization method before being fed into the networks.

Loss Function

The model was trained to minimize the negative partial log-likelihood, referred to as the Cox proportional hazards loss. This objective function quantifies the alignment between predicted log-risks and observed survival times. To ensure numerical stability, log-sum-exp tricks were applied during risk set computation, as delineated by the subsequent formula:

$$\mathcal{L}(\theta) = -\frac{1}{N_E} \sum_{i:\delta_i=1} \left(h_\theta(x_i) - \log \sum_{j \in R(T_i)} e^{h_\theta(x_j)} \right)$$

where: $h_\theta(x_j)$: Predicted log-risk for subject i, $R(T_i)$: Subjects at risk at time T_i, N_E: Number of observed events.

Hyperparameter Configuration

The network hyperparameters were strictly configured to optimize the survival prediction task:

Batch Size: 32

Maximum Epochs: 50

Optimizer: Stochastic Gradient Descent (SGD) was employed.

Learning Rate: The initial learning rate was set to 0.01. A cosine annealing scheduler was used across the 50 epochs to balance early-stage exploration and late-stage fine-tuning.

Regularization & Momentum: To prevent overfitting, Nesterov momentum (0.9) and weight decay (1e-4) were incorporated. Dropout (0.5) and batch normalization were applied after each convolutional block.

Training Strategy

The training protocol incorporated a warm-up phase during which the initial layers were frozen for 5 epochs to ensure the stability of feature extraction. Subsequently, a fine-tuning stage ensued, in which all layers were unfrozen. Gradient clipping (with a norm of 1.0) was employed to avert gradient explosion. Early stopping was executed by monitoring the validation loss with a patience of 10 epochs to capture the optimal model weights.

Table S1 The CT protocols of the 6 centers.

Parameters	Center 1	Center 2	Center 3	Center 4	Center 5	Center 6
CT version	Discovery CT750 HD (GE Healthcare, USA) or SOMATOM Definition Flash (Siemens Healthcare, Germany)	Aquilion One (Toshiba Medical Systems, Japan); LightSpeed VCT (GE Healthcare, USA)	Revolution CT (GE Healthcare, USA)	SOMATOM Definition AS (Siemens Healthcare, Germany); Revolution CT (GE Healthcare, USA)	Brilliance iCT (Philips Healthcare, Netherlands)	Brilliance iCT (Philips Healthcare, Netherlands)
CT tube voltage	100-120 kV	110-120 kV	100-120 kV	100-120 kV	120 kV	100–120 kV
CT tube current	200-500 mA	250-450 mA	200-500 mA	100-250 mA	250 mA	125–360 mA
Gantry rotation time	0.50-0.60 s	0.50s	0.28-0.33 s	0.28-0.33s	0.27s	0.27s
Detector collimation (mm)	0.625 mm	0.625 mm	0.625 mm	0.6-0.625 mm	0.625 mm	0.625 mm
Image matrix	512*512	512*512	512*512	512*512	512*512	512*512
Slice thickness	5-7 mm	1-7 mm	1-5 mm	1-5 mm	5 mm	1-7 mm

kV = kilovolt, mA = milliampere, mm = milimetre.

Table S2 C-indexes of different DL architectures in UTUC' OS prediction for the training set.

Network architecture	2D-DL	2.5D-DL
ResNet-50	0.681 (0.631-0.731)	0.705 (0.656-0.754)
Vgg-19	0.618 (0.566-0.671)	0.671 (0.621-0.722)
Densenet-121	0.620 (0.567-0.672)	0.672 (0.621-0.723)
Inception_v3	0.557 (0.503-0.610)	0.691 (0.642-0.741)

DL, deep learning.

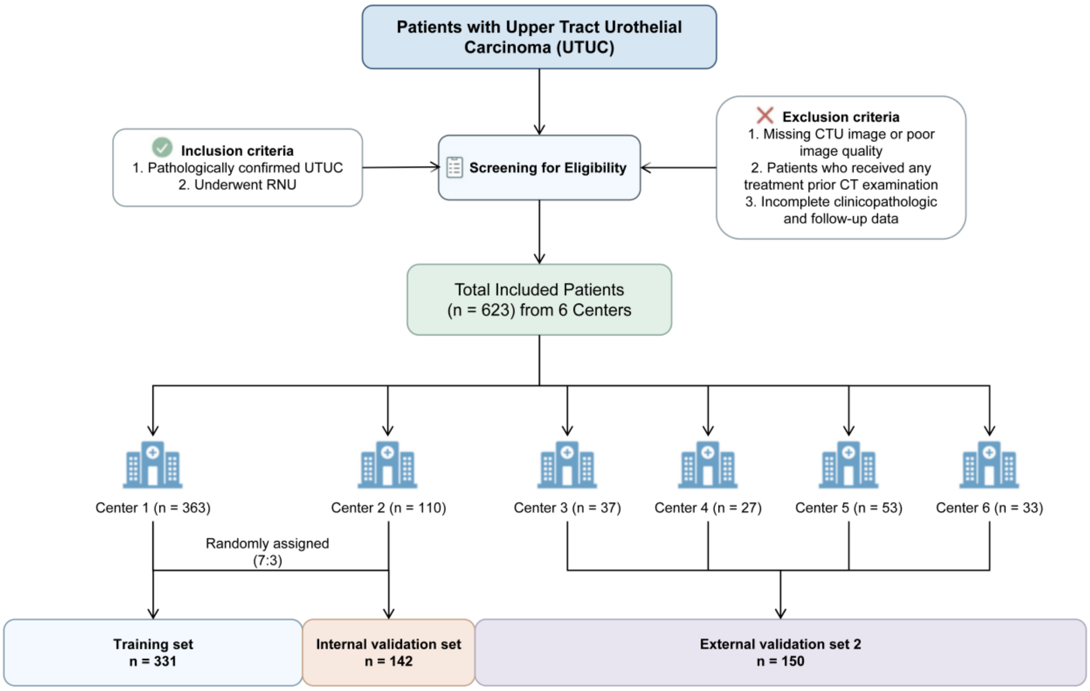

Figure S1. Patient enrollment and cohort distribution. A total of 623 patients with UTUC were included in the study from six different centers based on specific inclusion and exclusion criteria. UTUC, upper tract urothelial carcinoma.

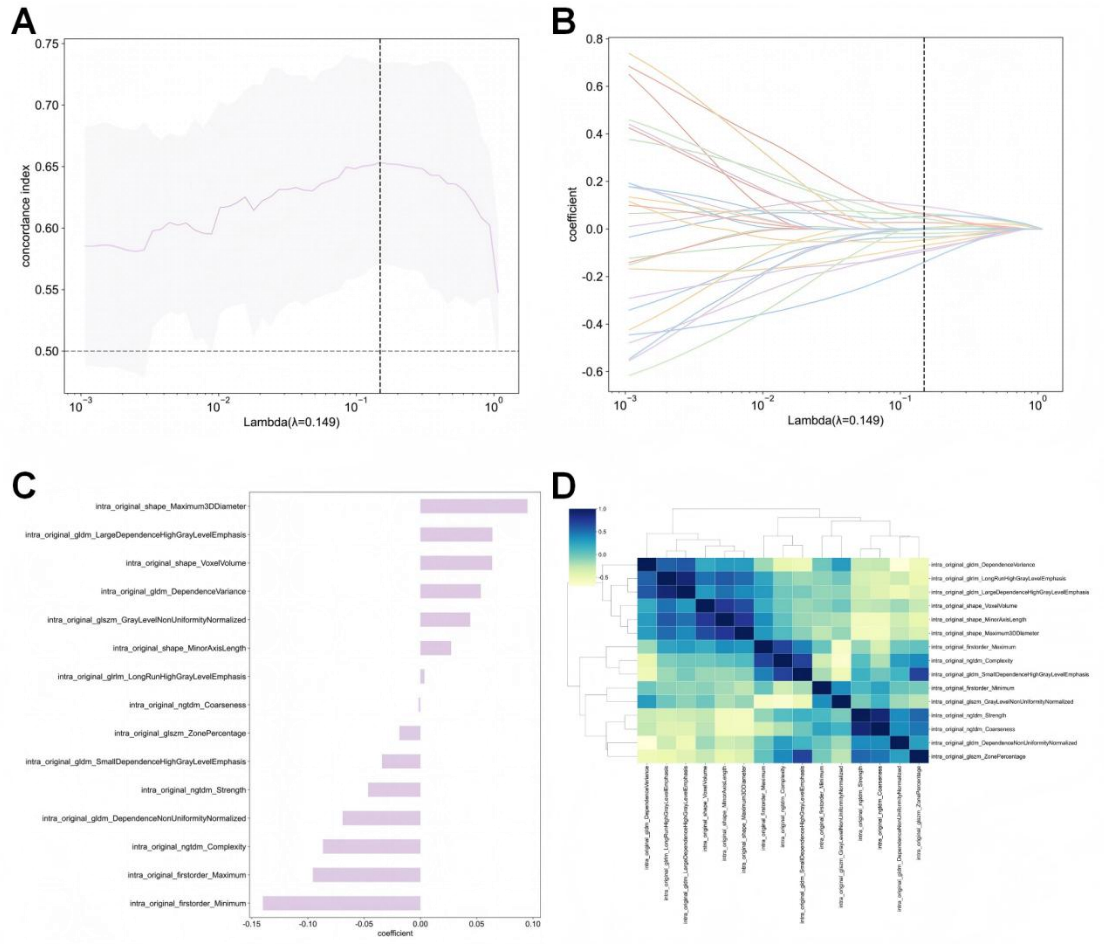

Figure S2. The process of radiomics feature selection. (A) A total of 15 significant radiomics features were identified and selected by the LASSO-Cox algorithm in Tuning parameter (λ) of 0.149. (B) A coefficient profile plot was generated versus the selected log λ value. (C) Feature weights plot and (D) correlation heatmap between radiomics features selected by LASSO regression algorithm. LASSO, least absolute shrinkage and selection operator.

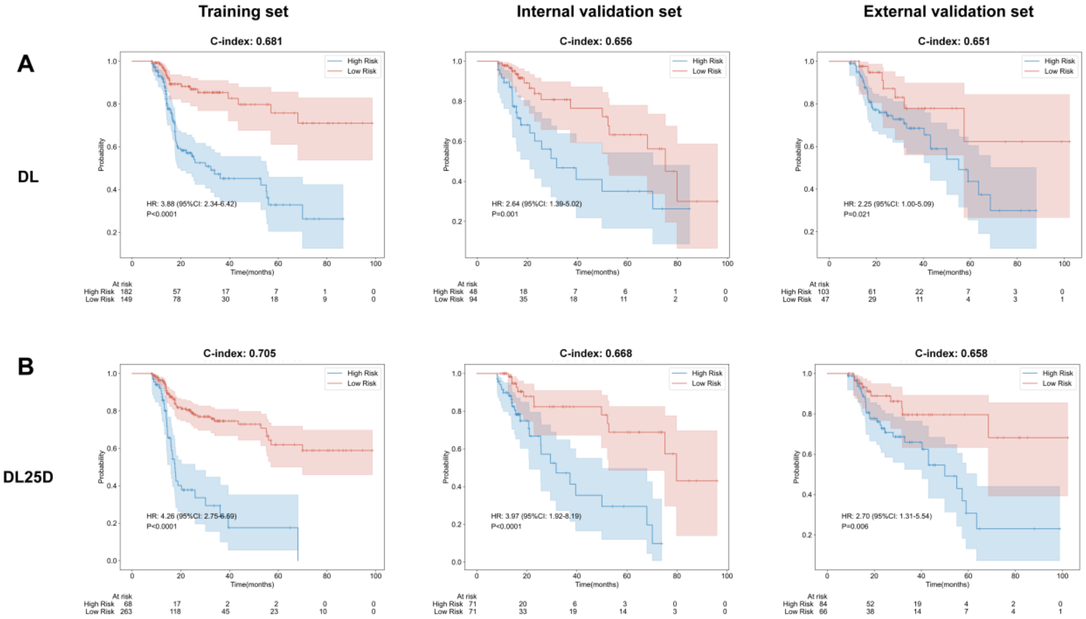

Figure S3. Prognostic value of the deep learning models. (A) KM survival curves for the DL Model are shown across the training set, internal validation set, and external validation set. (B) KM survival curves for the DL25D Model are shown across the same three cohorts. KM, Kaplan-Meier.

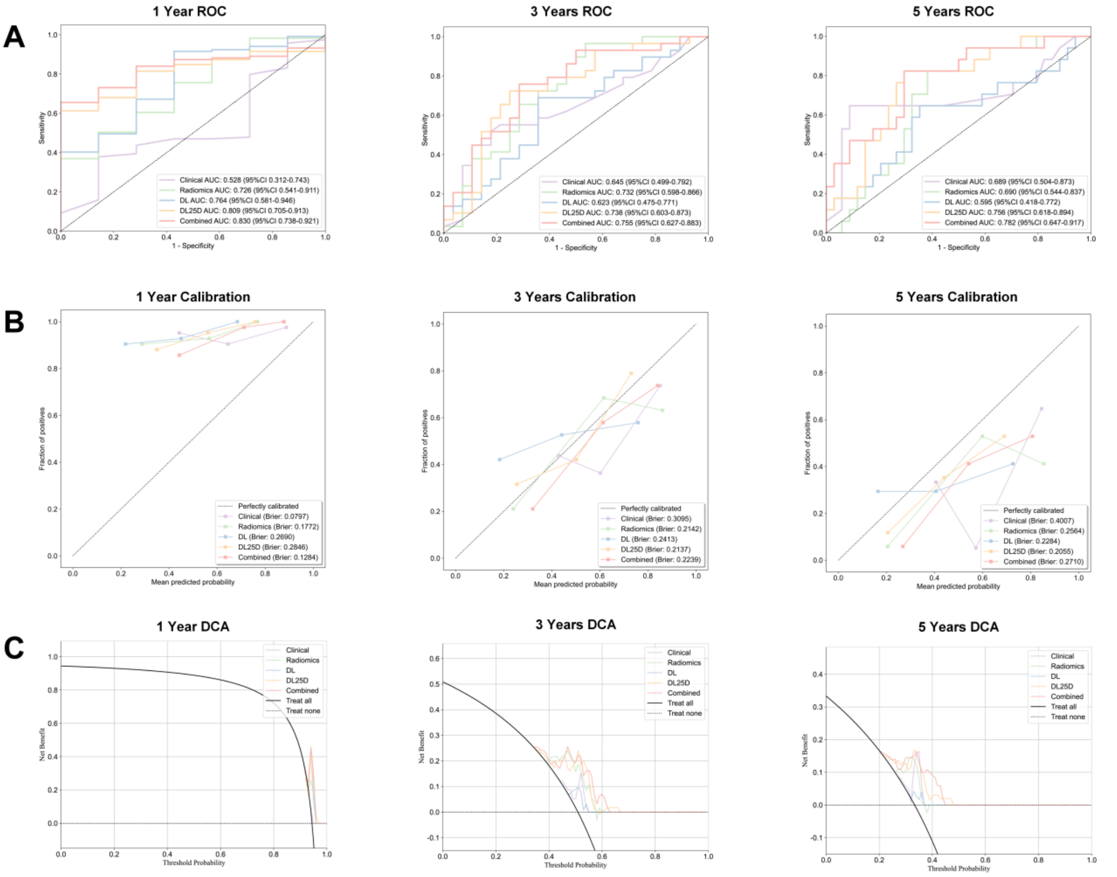

Figure S4. Model performance evaluation in the internal validation set. This figure shows the performance of the Clinical, Radiomics, DL, DL25D, and Combined models within the internal validation cohort. The performance of each model was evaluated at 1-, 3-, and 5-year follow-up intervals. (A) ROC curves are presented for all models. The AUC and 95% CI for each model are listed, demonstrating their discriminative ability. (B) Calibration curves are shown to assess the agreement between the predicted and actual survival probabilities. The dashed line represents a perfectly calibrated model. (C) DCA is presented to evaluate the clinical utility of each model. ROC, receiver operating characteristic, AUC, area under the ROC curve, CI, confidence interval, DCA, decision curve analysis.

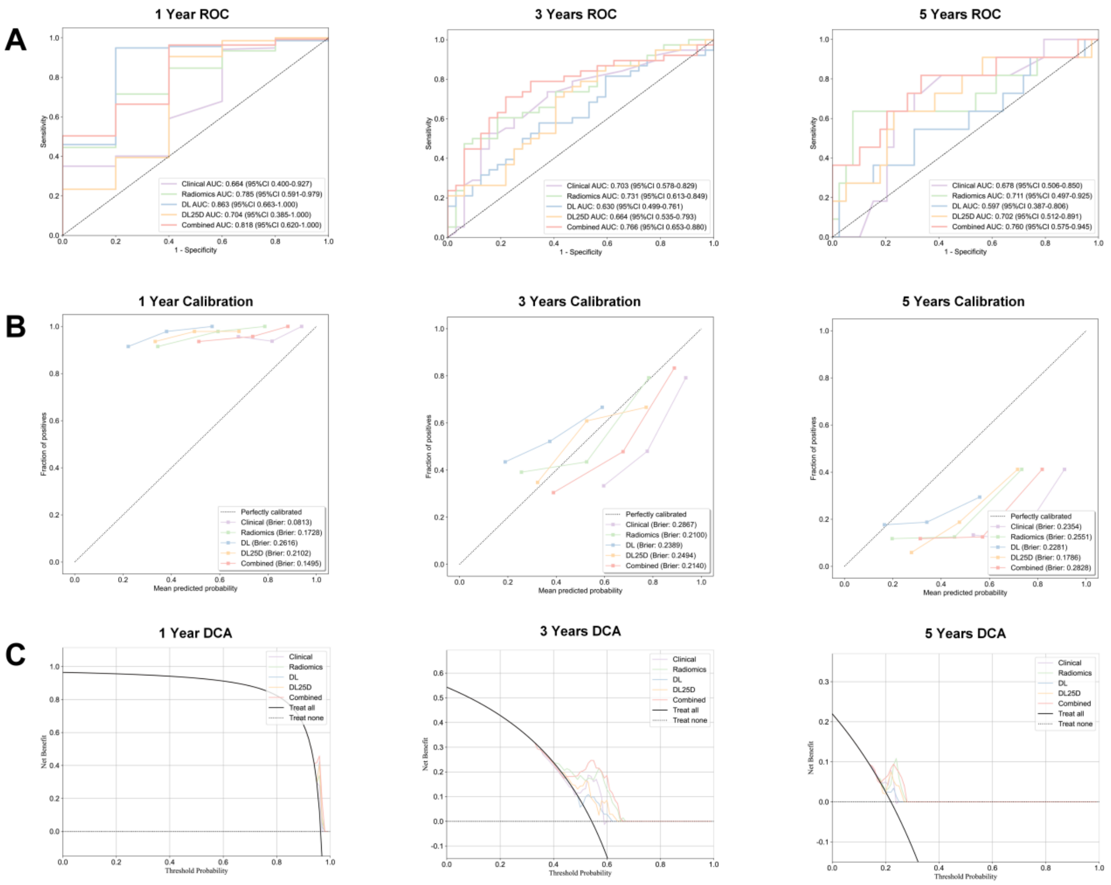

Figure S5. Model performance evaluation in the external validation set. This figure shows the performance of the Clinical, Radiomics, DL, DL25D, and Combined models within the external validation cohort. The performance of each model was evaluated at 1-, 3-, and 5-year follow-up intervals. (A) ROC curves are presented for all models. The AUC and 95% CI for each model are listed, demonstrating their discriminative ability. (B) Calibration curves are shown to assess the agreement between the predicted and actual survival probabilities. The dashed line represents a perfectly calibrated model. (C) DCA is presented to evaluate the clinical utility of each model. ROC, receiver operating characteristic, AUC, area under the ROC curve, CI, confidence interval, DCA, decision curve analysis.

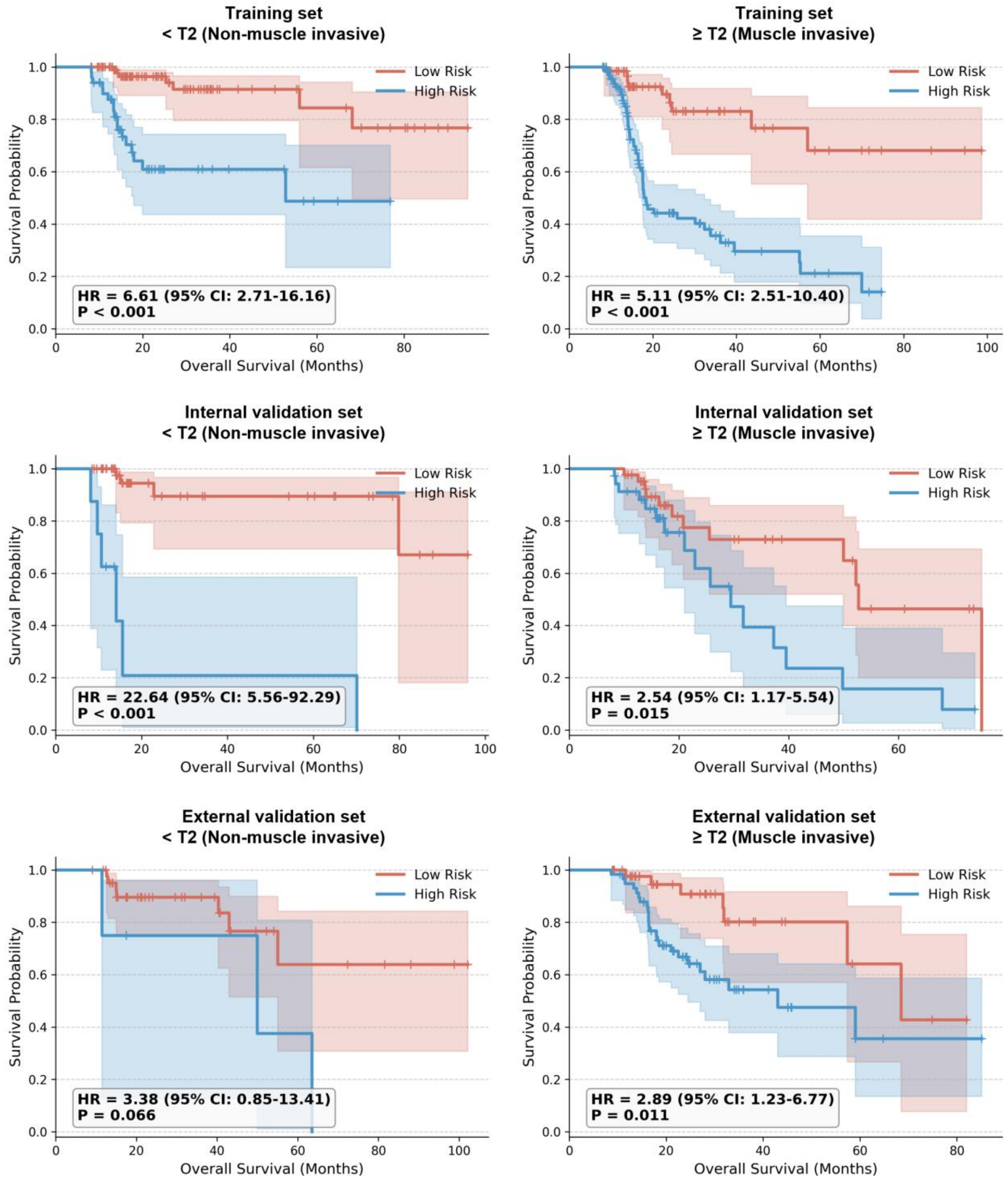

Figure S6. Kaplan-Meier survival curves of the combined model stratified by pathological T (pT) stage. Patients in the non-muscle invasive (<T2) and muscle-invasive (≥T2) subgroups across the training, internal validation, and external validation cohorts were stratified into high- and low-risk groups.
